# Association between female circulating heavy metal concentration and abortion: a systematic review and meta-analysis

**DOI:** 10.3389/fendo.2023.1216507

**Published:** 2023-08-29

**Authors:** Meiqi Ren, Liantong Wang, Liqin Wen, Jinghua Chen, Song Quan, Xiao Shi

**Affiliations:** ^1^ Center for Reproductive Medicine, Department of Obstetrics and Gynaecology, NanFang Hospital, Southern Medical University, Guangzhou, China; ^2^ The First School of Clinical Medicine, Southern Medical University, Guangzhou, China

**Keywords:** recurrent pregnancy loss, spontaneous abortion, endocrine dysfunction, zinc, copper, lead, cadmium

## Abstract

**Objective:**

This study aimed to evaluate the association between blood heavy metal (zinc (Zn), copper (Cu), lead (Pb), and cadmium (Cd)) concentrations and spontaneous abortion (SA) and recurrent pregnancy loss (RPL) and explore the possible endocrine dysfunction associated with it.

**Methods:**

A literature search was performed in the PubMed, Embase, Cochrane Library, and Web of Science databases up to April 2023. The overall effects were expressed as the standard mean difference (SMD). Subgroup analysis was performed according to the type of abortion (SA or RPL). Stata 16.0 was utilized for data analysis.

**Results:**

Based on the integrated findings, abortion women showed significantly lower Zn (SMD = −1.05, 95% CI: −1.74 to −0.36, *p* = 0.003) and Cu concentrations (SMD = −1.42, 95% CI: −1.97 to −0.87, *p <*0.001) and higher Pb (SMD = 1.47, 95% CI: 0.89–2.05, *p <*0.001) and Cd concentrations (SMD = 1.15, 95% CI: 0.45–1.85, *p* = 0.001) than normal pregnant women. Subgroup analysis showed that Zn and Cu deficiency and Cd and Pb exposure were significantly (*p <*0.05) associated with RPL, whereas Cu deficiency and Cd and Pb exposure were significantly (*p <*0.05) associated with SA.

**Conclusion:**

Zn and Cu deficiencies and Pb and Cd exposure were associated with abortion. Endocrine dysfunction, such as insulin resistance, vitamin D insufficiency, and abnormal thyroid and sex hormone concentrations, is thought to be involved in heavy metal-related abortion.

## 1 Introduction

Spontaneous abortion (SA) is a serious reproductive health problem with various definitions. According to the World Health Organization, SA is defined as the involuntary loss of a fetus weighing ≤500 g before the 20th gestational week (GW) (1), whereas the Chinese Medical Association Obstetrics and Gynecology Branch defines it as the involuntary loss of a fetus weighing ≤1,000 g before the 28th GW (2). SA occurs in 10%–15% of pregnancies, and approximately 80% of SA occurs before 12 weeks of pregnancy, which is known as early pregnancy loss (3). Recurrent pregnancy loss (RPL) is a special form of SA that affects 1.4% of women and causes physical and emotional challenges (4). However, the definition of RPL has been inconsistent. The European Society for Human Reproduction and Embryology (ESHRE) defines it as two or more abortions, irrespective of whether they are consecutive (5), while the American Society for Reproductive Medicine defines it as the loss of two or more consecutive pregnancies (6). There is controversy about the quantity and consecutiveness of abortions (7). The etiologies of SA and RPL, including chromosomal abnormalities, uterine malformations, and endocrine dysfunction, are complex (8–10). Exposure to environmental pollutants is also a risk factor for SA and RPL. Most pollutants are endocrine disrupters and early embryonic development is extremely sensitive to them (11, 12).

Heavy metals are among the most harmful environmental contaminants because they are not biologically degradable and can accumulate in organisms along the food chain (13). Heavy metals are mainly absorbed through air, drinking water, and contaminated food (14). They can be classified as essential (e.g., copper [Cu], zinc [Zn]) and non-essential (such as lead [Pb] and cadmium [Cd]). Essential metals play important roles in metabolism, enzymatic synthesis, and signal transduction, and their deficiency or overexposure may affect normal physiological functions of organisms (14). For instance, Zn and Cu are important components of several proteins, including antioxidant enzymes, metalloenzymes, and coenzymes, which are essential for fetal growth. Maternal Zn and Cu deficiency can reduce the fetal Zn and Cu supply through the placenta and cause fetal loss and pregnancy complications (15–17). Non-essential metals are usually toxic to humans, especially to human reproductive health, even at very low concentrations. Among all nonessential metals, Cd and Pb are endocrine-disrupting metals that can interfere with the production and secretion of sex hormones, leading to poor pregnancy outcomes (18).

Several previous studies have investigated the associations between the concentrations of Cd, Pb, Zn, and Cu in the blood and the risk of abortion (1, 19); however, the results have been inconsistent. Some studies have reported that exposure to heavy metals during early pregnancy can increase the incidence of SA and RPL (20–22), and endocrine dysfunction has been suggested as a mediator (23, 24) Other studies have reported contrasting findings (18). Given the increasing interest of clinicians and researchers, stronger evidence on the effect of heavy metal exposure on abortion and its underlying mechanisms is in demand. We performed the present meta-analysis to clarify the associations between abortion and the concentrations of Cd, Pb, Zn, and Cu. We also systematically reviewed the previous literature to explore the relationships between endocrine dysfunction, the four metals, and RPL or SA.

## 2 Methods

### 2.1 Study selection

The systematic review was conducted according to the Preferred Reporting Items for Systematic Reviews and Meta-Analyses (PRISMA) statement. PubMed, Embase, Cochrane Library, and Web of Science databases were searched for relevant studies published up to April 2023. The subject terms included ‘Miscarriage,’ ‘Pregnancy loss,’ ‘Abortion, Spontaneous,’ ‘Zinc,’ ‘Copper,’ ‘Lead,’ and ‘Cadmium.’ Random combinations of these subject terms and their synonyms were used for retrieval. The detailed literature search strategy is provided in the **Supplementary Material**. We reached the corresponding authors when the data were missing.

### 2.2 Inclusion criteria and exclusion criteria

Studies meeting the following criteria were included in the meta-analysis. (a) Study population: Pregnant women without internal and obstetric diseases that impair the normal process of pregnancy, including infectious diseases, gestational hypertension, gestational diabetes mellitus, and infertility. (b) Measurement: Female serum, plasma, or whole blood metal concentrations. (c) Observation group: Women who had experienced abortion, including SA and RPL. SA is defined as the involuntary loss of a fetus before the 28th GW (including the 20th and 24th GW) (2). RPL is defines as two or more abortions, irrespective of whether they are consecutive (5). (d) Control group: Healthy pregnant women with normal pregnancy or delivery. (e) Study type: Observational study.

The exclusion criteria were as follows: (a) article type: review, meta-analysis, meeting, case report, letter, comment, editorial, note, trial registry record, or protocol, (b) studies that focused on non-human cases (e.g., animal studies), (c) unclear definition of SA or RPL, (d) insufficient data on metal concentration; and (e) unavailable full text.

### 2.3 Quality assessment

The studies that met our inclusion criteria after the initial search were case–control, nested case–control, and cross-sectional studies. Therefore, the Newcastle-Ottawa Scale (NOS) was used to assess the quality of the studies (25). Each included article was independently appraised by two authors (MR and LiqW). Based on the NOS, studies were categorized as high- (8, 9), moderate- (6, 7), or low-quality (<6). Any disagreements regarding the assessment of the studies were discussed with the third author (LiaW).

### 2.4 Data extraction

Two investigators independently extracted the relevant data from the included studies (MR and LiaW). All data were double-checked by the third author (LiqW). The following information was extracted from the selected studies: first author, publication year, country and continent of the study population, type of detected sample, type of article, type of heavy metal, type of abortion, follow-up endpoint, sample size, concentrations of heavy metals, and analytical method employed.

### 2.5 Statistical analysis

Meta-analysis was performed using Stata 16.0 (Stata Corp, College Station, TX, USA). The standard mean difference (SMD) was adopted to integrate the data on metal concentration, as it is a continuous variable with different units across various studies. The 95% confidence intervals (CIs) were computed and presented as forest plots. For each study, statistical heterogeneity was assessed using Cochran’s Q-test and I^2^ statistics, and a random effects model was used to estimate the relationship between metal concentrations and abortion, as there was significant heterogeneity (*p <*0.05, I^2^ >50%). To investigate the impact of metal concentration on the different types of abortions (SA and RPL), a subgroup analysis was performed. To investigate the origin of the heterogeneity, four additional subgroup analyses were performed based on the follow-up endpoints (ongoing pregnancy and live birth) of participants, continent of the study population (Africa, Asia, North America, Oceania, and Europe), type of article (case–control study, cross-sectional study, and nested case–control study), and type of detected sample (serum, plasma, and whole blood). An influence analysis (sensitivity analysis) was conducted to improve the reliability of the meta-analysis results. A funnel plot and Begg’s and Egger’s tests were used to detect potential publication bias; *p*-values <0.05 represented significant statistical publication bias for Begg’s and Egger’s tests.

## 3 Results

### 3.1 Literature search

**Figure 1:**
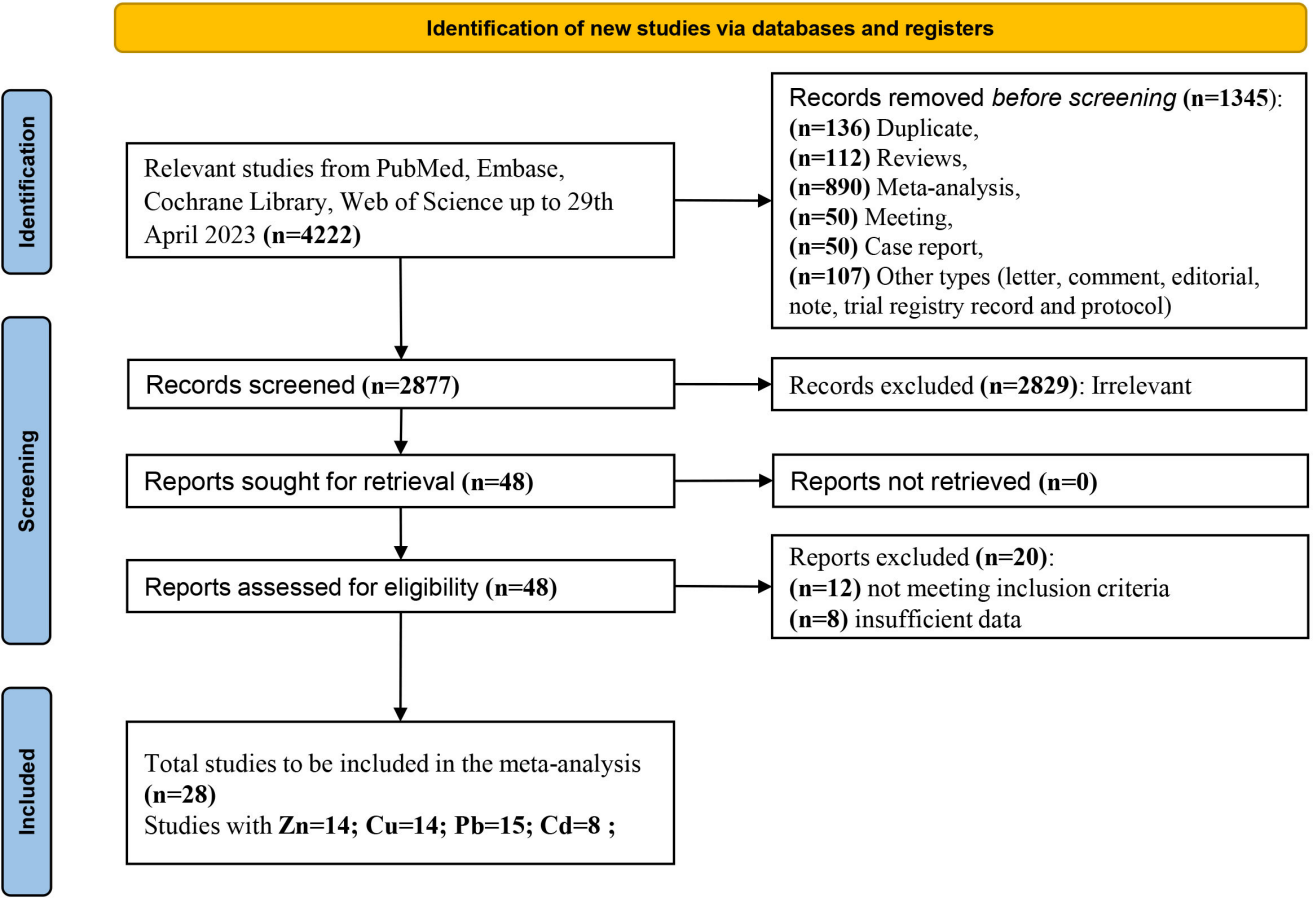
Flow chart of the included eligible studies in systematic review and meta-analysis.

**Figure 1** illustrates the PRISMA flow diagram for the selection of studies for inclusion in the systematic review and meta-analysis. A total of 4,222 potential studies were identified through database search. Among them, 136 articles were removed for duplicates, 1,209 articles were not observational studies (including reviews, meta-analyses, meetings, case reports, letters, comments, editorials, notes, trial registry records, and protocols), and 2,829 articles were not relevant to our study based on screening of their titles and abstracts by two authors. The literature screening results were double-checked to ensure that the relevant documents were not missed and did not need to be retrieved. After an independent review of the full texts by three authors (MR, LiaW, and LiqW), 12 studies were excluded because they did not meet the inclusion criteria, and eight studies were excluded because they had insufficient data (they only reported mean values without standard deviation of the metal concentration). Twenty-eight relevant studies were subjected to a final quantitative assessment based on the exclusion and inclusion criteria. Among the 28 studies, 14 investigated Zn and Cu, 15 investigated Pb, and eight investigated Cd.

### 3.2 Study characteristics

**Table 1** lists the baseline characteristics of the included articles. The included articles were observational studies published between 1979 and 2023 and involved 1,377 abortion cases (including 1,159 females with SA and 218 females with RPL), together with 3,289 normal pregnant females. Of the 28 articles included in this meta-analysis, six were completed in China (37, 38, 40, 42, 45, 51), three in Egypt (26, 30, 31), three in Saudi Arabia (29, 36, 47), three in Iran (48–50), two in Mexico (33, 39), two in Turkey (35, 52), two in Poland (19, 41), one in Nigeria (27), one in Croatia (28), one in Italy (32), one in Australia (34), one in Serbia (43), one in India (44), and one in Russia (46). The sample size of the included studies ranged from 38 to 1,447.

**Table 1:** Characteristics of the eligible studies.

										Number of subjects		Metal concentration (mg/dL)				
Sr.No	Author	Year	Country	Continent	Sample	Type of Article	Heavy Metal	Type of Abortion	Follow-up Endpoint	Cases	Controls	Cases	Controls	*p*	Analytical method employed	Reference
**1**	**Ahmed, M.H.**	**2007**	**Egypt**	**Africa**	**Serum**	**Case–control**	**Zinc**	**SA**	**12 weeks**	**24**	**14**	**0.76 ± 0.06 (mg/L)**	**0.86 ± 0.04 (mg/L)**	**0.000**	**AAS**	(26)
					**Serum**	**Case–control**	**Lead**	**SA**	**12 weeks**	**24**	**14**	**10.559 ± 1.317 (µg/dL)**	**7.977 ± 3 (µg/dL)**	**0.000**	**AAS**	
					**Serum**	**Case–control**	**Cadmium**	**SA**	**12 weeks**	**24**	**14**	**3.2 ± 0.65 (µg/L)**	**2.74 ± 0.25 (µg/L)**	**0.039**	**AAS**	
**2**	**Ajayi, O. O.**	**2012**	**Nigeria**	**Africa**	**Serum**	**Case–control**	**Zinc**	**RPL**	**20 weeks**	**35**	**34**	**99.25 ± 2.14 (μg/dL)**	**99.25 ± 2.14 (μg/dL)**	**0.001**	**AAS**	(27)
					**Serum**	**Case–control**	**Copper**	**RPL**	**20 weeks**	**35**	**34**	**94.25 ± 3.07 (μg/dL)**	**122.45 ± 2.71 (μg/dL)**	**0.001**	**AAS**	
					**Serum**	**Case–control**	**Lead**	**RPL**	**20 weeks**	**35**	**34**	**85.96 ± 1.09 (μg/dL)**	**60.70 ± 1.40 (μg/dL)**	**0.001**	**AAS**	
					**Serum**	**Case–control**	**Cadmium**	**RPL**	**20 weeks**	**35**	**34**	**4.58 ± 0.77 (μg/dL)**	**2.49 ± 0.09 (μg/dL)**	**0.001**	**AAS**	
**3**	**Alebic-Juretic, A.**	**2005**	**Croatia**	**Europe**	**Plasma**	**Case–control**	**Copper**	**SA**	**14weeks**	**17**	**28**	**18.2 ± 5.5 (μmol/L)**	**27.1 ± 7.6 (μmol/L)**	**<0.001**	**Photometry**	(28)
**4**	**Al-Sheikh, Y. A.**	**2019**	**Saudi**	**Asia**	**Plasma**	**Case–control**	**Zinc**	**RPL**	**Delivery**	**28**	**28**	**2.84 ± 0.36 (µmol/l)**	**3.55 ± 0.49 (µmol/l)**	**<0.001**	**ICP-MS**	(29)
					**Plasma**	**Case–control**	**Copper**	**RPL**	**Delivery**	**28**	**28**	**19.6 ± 2.75 (µmol/l)**	**24.5 ± 3.41 (µmol/l)**	**<0.001**	**ICP-MS**	
**5**	**Attalla, S.M.**	**2009**	**Egypt**	**Africa**	**Serum**	**Case–control**	**Zinc**	**RPL**	**12 weeks**	**40**	**24**	**77.01 ± 11.55 (µg%)**	**90.01 ± 10.77 (µg%)**	**0.020**	**AAS**	(30)
					**Serum**	**Case–control**	**Lead**	**RPL**	**12 weeks**	**40**	**24**	**19.78 ± 3.85 (µg/dL)**	**10.53 ± 1.01 (µg/dL)**	**<0.0001**	**AAS**	
					**Serum**	**Case–control**	**Cadmium**	**RPL**	**12 weeks**	**40**	**24**	**7.51 ± 1.02 (µg/dL)**	**5.06 ± 0.81 (µg/dL)**	**<0.0001**	**AAS**	
**6**	**Bassiouni, B. A.**	**1979**	**Egypt**	**Africa**	**Plasma**	**Case–control**	**Copper**	**SA**	**14 weeks**	**24**	**14**	**140.12 ± 15.20 (μg/100 mL)**	**188.57 ± 14.41 (μg/100 mL)**	**<0.01**	**AAS**	(31)
**7**	**Borella, P.**	**1990**	**Italy**	**Europe**	**Plasma**	**Case–control**	**Zinc**	**SA**	**16 weeks**	**12**	**41**	**13.64 ± 2.35 (μmol/L)**	**13.13 ± 2.37 (μmol/L)**	**>0.05**	**AAS**	(32)
					**Plasma**	**Case–control**	**Copper**	**SA**	**16 weeks**	**12**	**41**	**28.22 ± 4.68 (μmol/L)**	**26.05 ± 6.53 (μmol/L)**	**>0.05**	**AAS**	
**8**	**Borja-Aburto, V. H.**	**1999**	**Mexico**	**North America**	**Serum**	**Case–control**	**Lead**	**SA**	**20 weeks**	**35**	**60**	**12 ± 6.16 (μg/dL)**	**10.1 ± 5.34 (μg/dL)**	**0.021**	**AAS**	(33)
**9**	**Dreosti, I. E.**	**1990**	**Australia**	**Oceania**	**Serum**	**Case–control**	**Zinc**	**SA**	**12 weeks**	**35**	**37**	**0.75 ± 0.02 (μg/mL)**	**0.69 ± 0.02 (μg/mL)**	**>0.05**	**AAS**	(34)
					**Serum**	**Case–control**	**Copper**	**SA**	**12 weeks**	**35**	**37**	**1.36 ± 0.05 (μg/mL)**	**1.48 ± 0.05 (μg/mL)**	**>0.05**	**AAS**	
**10**	**Faikoğlu, R.**	**2006**	**Turkey**	**Asia**	**Serum**	**Case–control**	**Lead**	**SA**	**20 weeks**	**20**	**20**	**23.2 ± 1 3.77 (μg/dL)**	**18.04 ± 13.08 (μg/dL)**	**>0.05**	**AAS**	(35)
**11**	**Ghneim, H. K.**	**2016**	**Saudi**	**Asia**	**Plasma**	**Case–control**	**Zinc**	**RPL**	**Delivery**	**25**	**25**	**3.67 ± 0.39 (μmol/L)**	**4.11 ± 0.49 (μmol/L)**	**<0.001**	**ICP-MS**	(36)
					**Plasma**	**Case–control**	**Copper**	**RPL**	**Delivery**	**25**	**25**	**25.6 ± 3.25 (μmol/L)**	**28.8 ± 3.42 (μmol/L)**	**<0.001**	**ICP-MS**	
**12**	**Ghosh, A.**	**1985**	**China**	**Asia**	**Serum**	**Cross–section**	**Zinc**	**SA**	**12 weeks**	**45**	**55**	**120.18 ± 19.55 (μg/mL)**	**123.03 ± 18.57 (μg/mL)**	**>0.05**	**AAS**	(37)
**13**	**Jie, O.**	**2019**	**China**	**Asia**	**Whole blood**	**Case–control**	**Cadmium**	**SA**	**12 weeks**	**95**	**100**	**0.32 ± 0.28 (μg/L)**	**0.22 ± 0.11 (μg/L)**	**0.002**	**ICP-MS**	(38)
**14**	**Lamadrid-Figueroa, H.**	**2007**	**Mexico**	**North America**	**Plasma**	**Case–control**	**Lead**	**SA**	**12 weeks**	**71**	**136**	**0.14 ± 0.13 (µg/L)**	**0.13 ± 0.13 (µg/L)**	**0.15**	**ICP-MS**	(39)
**15**	**Lu, Y.**	**2022**	**China**	**Asia**	**Whole blood**	**Cross–section**	**Zinc**	**SA**	**12 weeks**	**92**	**103**	**5,082.32 ± 1,030.13 (µg/L)**	**5,243.88 ± 960.87 (µg/L)**	**0.251**	**ICP-MS**	(40)
					**Whole blood**	**Cross–section**	**Copper**	**SA**	**12 weeks**	**92**	**103**	**797.36 ± 161.42 (µg/L)**	**861.77 ± 188.75 (µg/L)**	**0.008**	**ICP-MS**	
					**Whole blood**	**Cross-section**	**Lead**	**SA**	**12 weeks**	**92**	**103**	**7.27 ± 3.01 (µg/L)**	**7.61 ± 2.64 (µg/L)**	**0.165**	**ICP-MS**	
**16**	**Omeljaniuk, W. J.**	**2015**	**Poland**	**Europe**	**Serum**	**Case–control**	**Zinc**	**SA**	**Delivery**	**83**	**35**	**0.5865 ± 0.1071 (mg/L)**	**0.6492 ± 0.1878 (mg/L)**	**>0.05**	**AAS**	(41)
					**Serum**	**Case–control**	**Copper**	**SA**	**Delivery**	**83**	**35**	**1.1532 ± 0.2980 (mg/L)**	**1.4450 ± 0.2930 (mg/L)**	**<0.002**	**AAS**	
**17**	**Omeljaniuk, W. J.**	**2018**	**Poland**	**Europe**	**Whole blood**	**Case–control**	**Lead**	**SA**	**Delivery**	**83**	**35**	**35.54 ± 11.0 (μg/L)**	**27.11 ± 4.6 (μg/L)**	**<0.0001**	**AAS**	(19)
					**Whole blood**	**Case–control**	**Cadmium**	**SA**	**Delivery**	**83**	**35**	**2.730 ± 2.07 (μg/L)**	**1.035 ± 0.59 (μg/L)**	**<0.0004**	**AAS**	
**18**	**Ou, J.**	**2020**	**China**	**Asia**	**Whole blood**	**Case–control**	**Lead**	**SA**	**12 weeks**	**150**	**150**	**27.21 ± 31.43 (μg/L)**	**15.96 ± 12.22 (μg/L)**	**0.000**	**ICP-MS**	(42)
**19**	**Popovic, J. K.**	**2016**	**Serbia**	**Europe**	**Plasma**	**Case–control**	**Copper**	**SA**	**12 weeks**	**35**	**50**	**20.52 ± 3.76 (μmol/L)**	**28.43 ± 4.45 (μmol/L)**	**<0.01**	**Colorimetry**	(43)
**20**	**Sairoz**	**2023**	**India**	**Asia**	**Serum**	**Nested Case–control**	**Zinc**	**SA**	**12 weeks**	**80**	**100**	**51.7 ± 10.4 (µg/dL)**	**81.6 ± 20.3 (µg/dL)**	**0.0000**	**Reaction with nitro-PAPS**	(44)
					**Serum**	**Nested Case–control**	**Copper**	**SA**	**12 weeks**	**80**	**100**	**222.5 ± 60.5 (µg/dL)**	**302.5 ± 95.2 (µg/dL)**	**0.0006**	**Reaction with Di-Br-PAESA**	
**21**	**Shen, P. J.**	**2015**	**China**	**Asia**	**Serum**	**Nested Case–control**	**Zinc**	**SA**	**12 weeks**	**58**	**1389**	**72.67 ± 11.98 (µmol/L)**	**83.25 ± 12.79 (µmol/L)**	**<0.05**	**AAS**	(45)
					**Serum**	**Nested Case–control**	**Copper**	**SA**	**12 weeks**	**58**	**1389**	**29.96 ± 5.27 (µmol/L)**	**31.24 ± 5.07 (µmol/L)**	**>0.05**	**AAS**	
**22**	**Skalnaya, M. G.**	**2019**	**Russia**	**Europe**	**Serum**	**Case–control**	**Copper**	**SA**	**28 weeks**	**75**	**169**	**1.12 ± 0.29 (µg/L)**	**1.60 ± 0.54 (µg/L)**	**<0.001**	**ICP-MS**	(46)
**23**	**Tabassum, H.**	**2022**	**Saudi**	**Asia**	**Serum**	**Case–control**	**Lead**	**RPL**	**12 weeks**	**30**	**30**	**77.96 ± 5.51 (ppb)**	**38.65 ± 0.20 (ppb)**	**<0.001**	**ICP-MS**	(47)
					**Serum**	**Case–control**	**Cadmium**	**RPL**	**12 weeks**	**30**	**30**	**0.45 ± 0.04 (ppb)**	**0.42 ± 0.01 (ppb)**	**<0.05**	**ICP-MS**	
**24**	**Tousizadeh, S.**	**2023**	**Iran**	**Asia**	**Serum**	**Case–control**	**Zinc**	**RPL**	**Delivery**	**60**	**60**	**5.26 ± 1.96 (mg/l)**	**15.06 ± 7.17 (mg/l)**	**<0.001**	**AAS**	(48)
					**Serum**	**Case–control**	**Lead**	**RPL**	**Delivery**	**60**	**60**	**3.69 ± 2.48 (mg/l)**	**0.31 ± 0.61 (mg/l)**	**<0.001**	**AAS**	
**25**	**Vigeh, M.**	**2010**	**Iran**	**Asia**	**Whole blood**	**Case–control**	**Lead**	**SA**	**Delivery**	**15**	**336**	**3.51 ± 1.42 (μg/dL)**	**3.83 ± 1.99 (μg/dL)**	**0.41**	**ICP-MS**	(49)
**26**	**Vigeh, M.**	**2021**	**Iran**	**Asia**	**Whole blood**	**Case–control**	**Lead**	**SA**	**Delivery**	**25**	**141**	**55.43 ± 54.3 (µg/L)**	**44.97 ± 45.6 (µg/L)**	**0.307**	**ICP-MS**	(50)
					**Whole blood**	**Case–control**	**Cadmium**	**SA**	**Delivery**	**25**	**141**	**0.51 ± 0.5 (µg/L)**	**0.51 ± 0.5 (µg/L)**	**0.957**	**ICP-MS**	
**27**	**Wang, R.**	**2020**	**China**	**Asia**	**Serum**	**Cross–section**	**Zinc**	**SA**	**12 weeks**	**56**	**55**	**4.18 ± 0.26 (mg/L)**	**3.24 ± 1.47 (mg/L)**	**>0.05**	**ICP-MS**	(51)
					**Serum**	**Cross–section**	**Copper**	**SA**	**12 weeks**	**56**	**55**	**1.80 ± 0.58 (mg/L)**	**1.41 ± 0.55 (mg/L)**	**<0.001**	**ICP-MS**	
					**Serum**	**Cross–section**	**Lead**	**SA**	**12 weeks**	**56**	**55**	**0.17 ± 0.09 (mg/L)**	**0.15 ± 0.10 (mg/L)**	**>0.05**	**ICP-MS**	
**28**	**Yildirim, E.**	**2019**	**Turkey**	**Asia**	**Whole blood**	**Case–control**	**Lead**	**SA**	**12 weeks**	**29**	**20**	**54.11 ± 17.27 (µ/L)**	**44.45 ± 12.49 (µ/L)**	**0.038**	**AAS**	(52)
					**Whole blood**	**Case–control**	**Cadmium**	**SA**	**12 weeks**	**29**	**20**	**0.39 ± 0.06 (µ/L)**	**0.40 ± 0.05 (µ/L)**	**0.704**	**AAS**	

SA, Spontaneous Abortion; RPL, Recurrent Pregnancy Loss; AAS, Atomic-Absorption Spectrophotometry; ICP-MS, Inductively Coupled Plasma Mass spectrophotometry.

### 3.3 Quality of included studies

The quality assessment results for all studies are shown in **Supplementary Table 1**. Studies with quality scores higher than 6, which is the cut-off NOS score for low quality, were considered credible. All of the included articles had quality scores above 6.

### 3.4 Meta-analysis for Zn

**Figure 2:**
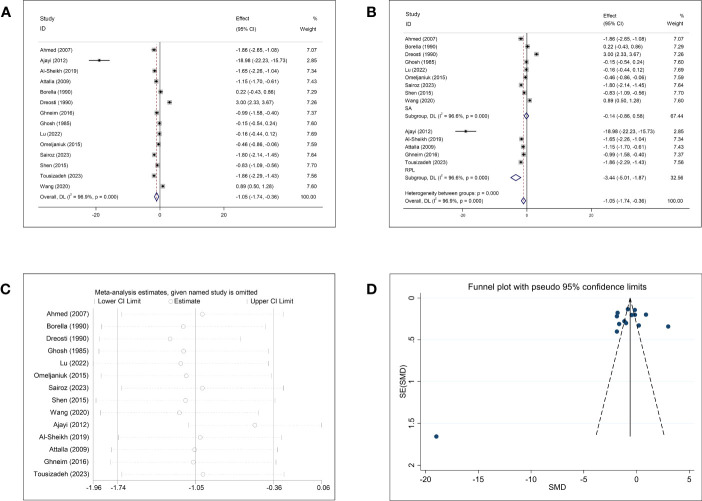
Meta-analysis outcomes of zinc. **(A)** Forest plot showing the meta-analysis outcomes between abortion group and normal pregnant women; **(B)** Subgroup analysis based on the type of abortion (SA and RPL); **(C)** Sensitivity analysis; and **(D)** Funnel plot.

Fourteen studies investigated the association between Zn concentrations and abortion. The pooled effect size showed that the Zn concentration was negatively associated with abortion (SMD = −1.05, 95% CI: −1.74, −0.36, *p* = 0.003, I^2^ = 96.9%; **Figure 2A**). Subgroup analysis showed that women with RPL had significantly lower Zn concentrations than healthy controls (SMD = −3.44, 95% CI: −5.01 to −1.87, *p <*0.001), whereas the Zn concentrations of women with SA and healthy controls were not significantly different (SMD = −0.14, 95% CI: −0.86–0.58, *p* = 0.710). Significant heterogeneity was observed in each subgroup (SA, *p <*0.001, I^2^ = 96.6%; RPL, *p <*0.001, I^2^ = 96.6%; **Figure 2B**). To investigate the origin of the high heterogeneity, subgroup analyses based on follow-up endpoint, continent, type of article, and type of detected sample were performed. Subgroup analyses revealed persistently high heterogeneity (**Supplementary Figure 1**). Sensitivity analysis showed that omission of any study did not change the overall effect (**Figure 2C**). There was no evidence of publication bias among the included studies (Begg, *p* = 0.511; Egger, *p* = 0.335; **Figure 2D**).

### 3.5 Meta-analysis for Cu

**Figure 3:**
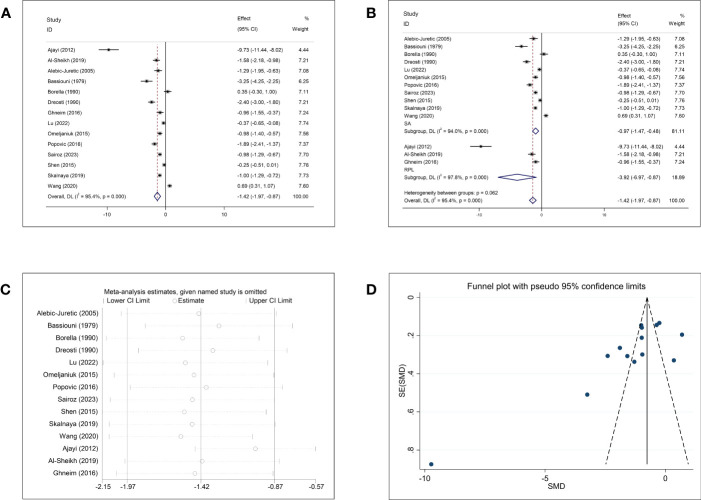
Meta-analysis outcomes of copper. **(A)** Forest plot showing the meta-analysis outcomes between abortion group and normal pregnant women; **(B)** Subgroup analysis based on the type of abortion (SA and RPL); **(C)** Sensitivity analysis; and **(D)** Funnel plot.

Comparisons of Cu concentrations in women with and without abortion were reported in 14 studies. The pooled effect size of the 14 studies revealed significantly lower Cu concentrations in the abortion group than in the control group (SMD = −1.42, 95% CI: −1.97, −0.87, *p <*0.001, I^2^ = 95.4%) (**Figure 3A**). Subgroup analysis stratified by the type of abortion (SA and RPL) showed that patients with SA and RPL had lower Cu concentrations than the healthy controls (**Figure 3B**); the SMD was −0.97 (95% CI: −1.47, −0.48, *p <*0.001) for women with SA and −3.92 (95% CI: −6.97, −0.87, *p* = 0.012, respectively). However, subgroup analysis based on abortion type showed obvious heterogeneity (SA, *p <*0.001, I^2^ = 94.0%; RPL, *p <*0.001, I^2^ = 97.8%). Subgroup analyses for the follow-up endpoint, continent, type of article, and type of detected sample showed high heterogeneity (**Supplementary Figure 2**). Sensitivity analysis showed that omission of any single study did not change the overall effect (**Figure 3C**). Publication bias was detected in the studies that included Cu (Begg: *p* = 0.037; Egger: *p* = 0.012). Visual inspection of funnel plots showed asymmetry (**Figure 3D**).

### 3.6 Meta-analysis for Pb

**Figure 4:**
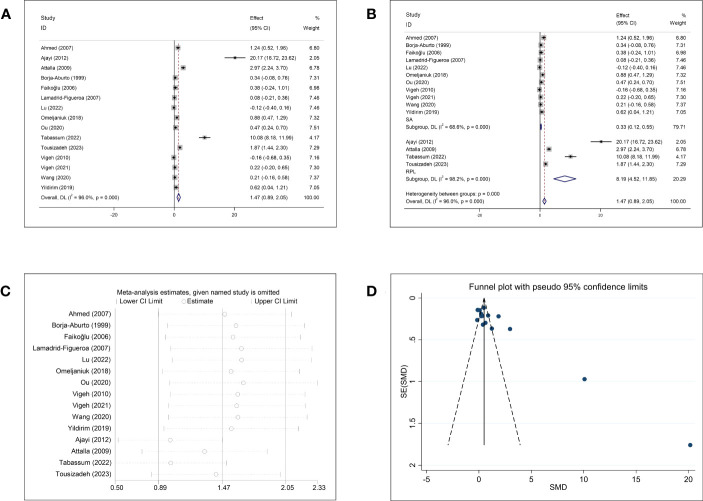
Meta-analysis outcomes of lead. **(A)** Forest plot showing the meta-analysis outcomes between abortion group and normal pregnant women; **(B)** Subgroup analysis based on the type of abortion (SA and RPL); **(C)** Sensitivity analysis; and **(D)** Funnel plot.

The meta-analysis of the association between Pb concentration and abortion included 15 studies (**Figure 4A**). The pooled circulating Pb concentration was significantly higher in women who had experienced an abortion than in those with normal pregnancies (SMD = 1.47, 95% CI: 0.89–2.05, *p <*0.001, I^2^ = 96.0%). Subgroup analysis for SA and RPL showed significantly higher Pb concentrations in women with SA (SMD = 0.33, 95% CI: 0.12–0.55, *p* = 0.002) and RPL (SMD = 8.19, 95% CI: 4.52–11.85, *p <*0.001) than in healthy pregnant women (**Figure 4B**). However, significant heterogeneity was observed (SA: *p <*0.001, I^2^ = 68.6%; RPL: *p <*0.001, I^2^ = 98.2%). Further subgroup analyses based on the follow-up endpoint, continent, type of article, and type of detected sample also showed high heterogeneity (**Supplementary Figure 3**). Sensitivity analysis showed that omission of any single study did not change the overall effect (**Figure 4C**). Visual inspection of the funnel plots (**Figure 4D**) and Begg’s and Egger’s tests showed publication bias (Begg: *p* = 0.002; Egger: *p* = 0.001).

### 3.7 Meta-analysis for Cd

**Figure 5:**
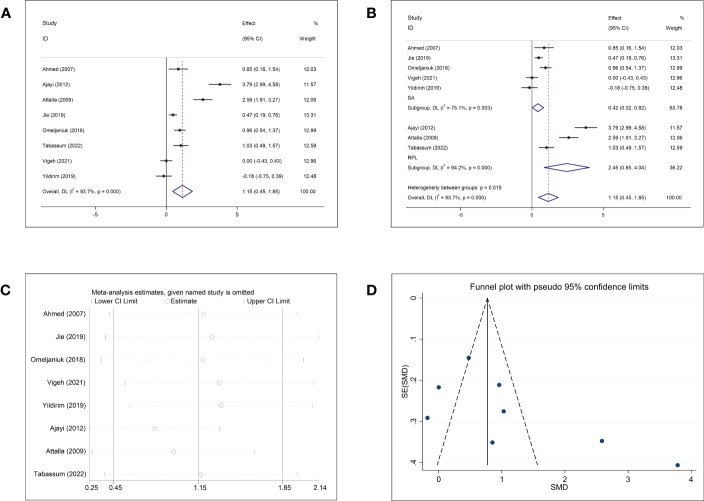
Meta-analysis outcomes of cadmium. **(A)** Forest plot showing the meta-analysis outcomes between abortion group and normal pregnant women; **(B)** Subgroup analysis based on the type of abortion (SA and RPL); **(C)** Sensitivity analysis; and **(D)** Funnel plot.

The pooled results of the meta-analysis of eight studies on Cd and abortion showed significantly higher Cd concentrations in women who underwent abortion than in normal pregnant women (SMD = 1.15, 95% CI: 0.45–1.85, *p* = 0.001, I^2^ = 93.7%) (**Figure 5A**). Subgroup analysis based on abortion type showed that women with SA and RPL had significantly higher Cd concentrations than the normal controls (SA: SMD = 0.42, 95% CI: 0.02–0.82, *p* = 0.040; RPL: SMD = 2.45, 95% CI: 0.85–4.04, *p* = 0.003). However, the heterogeneity was significant in each subgroup (SA: *p* = 0.003, I^2^ = 75.1%; RPL: *p <*0.001, I^2^ = 94.2%) (**Figure 5B**). Further subgroup analyses based on the follow-up endpoint, continent, and type of detected sample also showed high heterogeneity (**Supplementary Figure 4**). All studies on Cd and abortion were case–control studies, and subgroup analysis for different article types could not be performed. The sensitivity analysis showed that the exclusion of any single study could change the overall effect (**Figure 5C**). Both funnel plots and Begg’s and Egger’s tests showed no publication bias for Cd (Begg: *p* = 0.174; Egger: *p* = 0.113; **Figure 5D**).

## 4 Discussion

Due to widespread human exposure to (53) and bio-accumulation of heavy metals (54), there are growing concerns about the adverse effects of heavy metals on normal pregnancies. Exposure to toxic metals or deficiency of essential metals has long been suspected to lead to abortion (55). However, the results of previous studies have not been consistent (1, 19). To provide stronger evidence for this important clinical issue, we conducted the present meta-analysis, focusing on two common toxic metals (Pb and Cd) and two essential metals (Zn and Cu) (56). This study is first to investigate the overall association between blood Zn, Cu, Pb, and Cd concentrations and abortion, including RPL and SA. Zn or Cu deficiency was associated with the prevalence of abortion in women, and exposure to Pb or Cd increased the risk of abortion (SA and RPL). Only one relevant meta-analysis was carried out in 2021, showed that exposure to Cd and Pb increased the incidence of abortion (undistinguished threatened abortion, SA, and RPL) (23). Subgroup analysis based on abortion type was not performed (23). In the present study, we recruited more studies to reinforce the association between exposure to Cd and Pb and the increased risk of abortion and performed subgroup analysis based on the type of abortion to investigate the effect of Cd and Pb exposure on patients with SA and RPL. In addition, the exploration of Zn and Cu in women with RPL and SA provides a basis for clinicians who tend to intervene early against RPL in women with Zn and Cu deficiencies.

The exact mechanisms underlying the induction of SA and RPL by Pb and Cd exposure and Zn and Cu deficiency are unknown. Studies have shown that heavy metals are common environmental endocrine disruptors. Previous studies have reported that exposure to toxic metals and deficiencies of essential metals lead to abortion mainly through endocrine dysfunction, such as insulin resistance, vitamin D insufficiency, and abnormal thyroid and sex hormone concentrations, among others. The details of this process are discussed below.

### 4.1 Female Zn concentration and abortion

In our study, we found that Zn inadequacy tended to increase the chances of abortion, especially for RPL; however, it may not increase the incidence of SA. The underlying mechanism of Zn inadequacy-related RPL remains unknown. However, it may also be associated with endocrine dysfunction caused by Zn deficiency.

Zn deficiency has been reported to decrease insulin sensitivity and cause insulin resistance (IR) (57, 58), whereas Zn supplements can decrease IR (59, 60). IR, defined clinically as a decreased biological response to exogenous or endogenous insulin, can cause mitochondrial dysfunction in the placenta, diminished trophoblast invasion, a subclinical inflammatory state, and oxidative stress. These factors are all considered crucial in the pathophysiology of RPL (61–64). Zn can reinforce glucose transport into cells and potentiate insulin-induced glucose transport via the insulin signaling pathway (65). Zn can also act as an insulin mimetic to maintain glucose homeostasis, which may also be a mechanism underlying Zn deficiency-induced IR (66).

Apart from IR, Zn deficiency is also closely related to vitamin D deficiency, as Zn regulates the transcriptional activation of hormone-related genes via a cysteine-rich Zn-finger region in vitamin D receptors (VDRs) (67–69). Vitamin D plays a vital role in maintaining normal biological functions, such as calcium homeostasis, and cell proliferation, differentiation, and apoptosis, all of which are crucial for immunomodulation and normal pregnancy (70). Vitamin D inadequacy was reported to be associated with SA and RPL in a recent meta-analysis (71, 72). Supplementation with vitamin D can suppress inflammatory cytokine production and elevate the secretion of cathelicidin in decidual cells and trophoblasts, which can reduce the risk of abortion (73–75).

Zn deficiency appears to interfere with sex hormone synthesis and further causes RPL. Zn can affect the biosynthesis and function of sex hormones, such as progesterone and prolactin, by altering LH and FSH levels and inducing oxidative stress (17, 76–78). Zn may also promote estrogen release by forming ligand bonds with metal-binding sites on the estrogen receptor (ER) (79). Insufficient secretion of sex hormones, such as progesterone, testosterone, estrogen, and prolactin, can reduce endometrial receptivity and oocyte quality in women, which is related to RPL (55, 80, 81).

Furthermore, Zn is an essential trace element for thyroid function and homeostasis (82), and its deficiency can lead to hypothyroidism (82–84). Hypothyroidism and subclinical hypothyroidism can lead to poor pregnancy outcomes such as SA and RPL (85, 86). Zn supplements can elevate thyroxine (T4) concentrations and reduce triiodothyronine (T3) concentrations by altering the expression of key genes (*nis*, *tpo*, *thrα*, *dio1*, *dio2*, and *ugt1ab*) in the hypothalamic–pituitary–thyroid (HPT) axis (87).

### 4.2 Female Cu concentration and abortion

We found that women undergoing abortion (both SA and RPL) had lower Cu concentrations, indicating that Cu deficiency may be closely related to the incidence of abortion (SA and RPL). However, the underlying mechanism remains unknown. However, previous studies have reported that Cu deficiency can induce endocrine dysfunction, such as IR, vitamin D insufficiency, and abnormal thyroid and sex hormone concentrations, which may be involved in the pathogenesis of SA and RPL. Insufficient Cu can cause IR by upregulating cytochrome c oxidase 1 (SCO1) and vascular adhesion protein-1 (VAP-1) (88–90); it can also reduce progesterone synthesis by regulating the expression of steroidogenic factor 1 (SF-1) (91). Cu deficiency can also reduce Cu/Zn superoxide dismutase (Cu/Zn-SOD) and cause oxidative stress in the ovary, ultimately leading to dysfunctional luteal formation and insufficient progesterone secretion (17). In addition, Cu deficiency can decrease the expression of estrogen synthetases such as aromatase (CYP19A1) and 17β-hydroxysteroid dehydrogenase (17β-HSD) (92). Furthermore, Cu deficiency can lead to hypothyroidism (82–84, 93) by inducing oxidative stress and decreasing thyroxine synthesis by limiting tyrosinase availability (82, 93–95).

### 4.3 Female Pb concentration and abortion

Our study found that women who experienced abortion (SA and RPL) had higher Pb concentrations, suggesting that Pb exposure could increase the risk of abortion (SA and RPL). Our results are consistent with those of the meta-analysis by Kaur et al. (23). Pb can substitute polyvalent cations, such as calcium (Ca^2+^), and affect various cellular processes, such as apoptosis, cell adhesion, and cell signaling (96). However, the mechanism underlying Pb-induced abortion remains unclear. Animal studies have shown that Pb exposure can downregulate IR-related genes in the P13K and Akt signaling pathways, which are involved in hepatic gluconeogenesis and glucose production (97, 98). Low-level Pb exposure promotes the gene expression of key enzymes involved in hepatic gluconeogenesis and eventually induces hyperglycemia and impaired fasting plasma glucose, which is known as hepatic insulin resistance (99). Additionally, Pb appears to be involved in the pathology of vitamin D deficiency. Pb can diminish the activity of vitamin D by blocking the normal renal synthesis of active 1,25-dihydroxy vitamin D (1,25(OH)2D) and reduce the generation of vitamin D binding protein (DBP) (56, 100, 101). Pb can also promote degradation and block the synthesis of 1,25(OH)2D3 by upregulating the hepatic expression of Cyp24a1 enzymes and inhibiting 25-hydroxylase (CYP2R1) and 1-α-hydroxylase (CYP27B1) at the gene and protein levels (100, 102). Previous studies have suggested that Pb may be closely associated with luteal phase deficiencies. Pb can directly inhibit the expression of several key enzymes involved in progesterone synthesis, such as StAR, CYP11A1, and 3β-HSD (103, 104). Pb also appears to indirectly interfere with progesterone synthesis by inhibiting the cAMP-PKA-dependent signaling pathway that regulates the expression of these key enzymes (104–106). Pb has adverse effects on sex hormone concentration. Pb exposure is associated with increased testosterone and prolactin concentrations and appears to reduce estrogen concentrations by decreasing the expression of estrogen synthases such as 17β-HSD (103, 107, 108). Furthermore, Pb accumulation negatively affects thyroid function, which is also related to abortion. Excessive exposure to Pb may lead to hypo- or hyperthyroidism (109). As an oxidant, Pb can negatively impact thyroid cells by promoting oxidative stress, and it can also interact with other essential elements such as Cu, Zn, and Fe to indirectly affect thyroid function (82, 109).

### 4.4 Female Cd concentration and abortion

We found that the Cd concentration was significantly higher in women who experienced abortion (SA and RPL) than in normal pregnant women. Our results are in line with those of Kaur et al., who revealed that Cd exposure could increase the risk of abortion (23). Cd is a highly potent environmental pollutant that causes indirect oxidative damage to DNA, leading to the induction of cellular proliferation and inhibition of DNA repair mechanisms, causing cytotoxicity (110). However, research on the mechanisms of Cd exposure-related abortion is lacking. In recent years, an increasing number of studies have found a strong relationship between Cd and endocrine dysfunction, which is the main reason for abortions (both SA and RPL). Epidemiological surveys have shown that Cd can cause IR through perturbations in gluconeogenesis, pancreatic islet dysfunction, and metabolic and mitogen impairments in the liver and adipose tissue (111, 112). Epidemiological studies have also demonstrated that high blood Cd concentrations are negatively correlated with vitamin D concentrations (113, 114), which may be due to the interaction of Cd with renal mitochondrial hydroxylases (115). Cd is also involved in the pathogenesis of luteal phase deficiencies. It can directly or indirectly inhibit the expression of several key enzymes (StAR, CYP11A1, and 3β-HSD) involved in progesterone synthesis by regulating the cAMP-PKA-dependent signaling pathway (24, 116, 117). Cd can also interfere with the balance of sex hormone concentrations. Cd exposure appears to decrease the expression of estrogen synthetases (CYP19A1 and 17β-HSD), and it is also a potent xenoestrogen that can mediate the proliferation of anterior pituitary cells and prolactin secretion by mimicking estrogen (118). Cd can also negatively affect thyroid cells by promoting oxidative stress, ultimately leading to thyroid dysfunction (82, 109).

### 4.5 Strengths

The strength of our study is that we comprehensively investigated the relationships between blood Zn, Cu, Pb, and Cd concentrations and abortion rates (SA and RPL). In addition, we systematically reviewed previous publications on the endocrine mechanisms of metal exposure-related abortions. We propose that IR, vitamin D insufficiency, and abnormal thyroid and sex hormone concentrations may be involved in Zn and Cu deficiencies and Pb and Cd exposure-associated abortions. Third, the large sample size of 4,666 pregnant women from 14 countries makes our estimates reliable. Fourth, most included studies were of good quality. In the included studies, the definitions of cases and controls were adequate, and the selection of controls and assessment of exposure were consistent. Fifth, a sensitivity analysis was conducted to verify the associations between the four metals and abortion.

### 4.6 Limitations

This study has some limitations. First, several dated documents that appeared to meet our inclusion criteria were not included because we were unable to reach the authors. Second, despite our best efforts, we were only able to find 28 related papers because of the relatively large number of animal studies and case reports. Third, most meta-analyses included in our study had high heterogeneity. To ascertain its sources, we performed a subgroup analysis based on the type of abortion, follow-up endpoint, continent, type of observational study, and type of detected sample. However, we failed to find sources of heterogeneity by subgroup analysis, as most subgroup analyses showed high heterogeneity. After carefully reviewing the included articles, we found that different diagnoses of SA or RPL may have led to clinical heterogeneity. In addition, regional variations in metal concentrations of the study participants were considered another source of heterogeneity. Local mineral deposits and their exploitation affect the metal concentrations in the environment (air, water, and soil), and different terrains can impact the diffusion of pollutants (119). Thus, participants had different risks of metal exposure. Moreover, participants of different races with different genetic backgrounds have various sensitivities to metal exposure (120). Owing to the limited information regarding the region and race of the investigated subjects in the original literature, we were only able to perform a subgroup analysis based on different continents to investigate the heterogeneity caused by regional differences. The age of the study population and the time point of blood collection in each study were also considered potential sources of heterogeneity, as heavy metals could accumulate in the human body, and older adults may have higher blood metal concentrations. Furthermore, metal concentrations can change during different trimesters and the time points of blood collection may lead to heterogeneity (121, 122). Fourth, the literature regarding Cu and Pb had obvious publication and reporting bias, although the publication and reporting the bias of literature regarding Zn and Cd were acceptable. Language and multiple publication biases were considered primary problems as only the English literature was included, and two studies had outcomes from the same study population.

### 4.7 Implications for treatment

The findings of the present study broaden our understanding of the effects of toxic and essential metals on the RPL and SA. Endocrine dysfunction can lead to metal exposure and abortions. It will be helpful to screen blood Zn, Cu, Pb, and Cd concentrations in females. However, well-designed prospective cohort studies are needed to clarify the causal relationship between endocrine dysfunction and heavy-metal-induced abortion.

## 5 Conclusion

In the present study, we found that higher blood Pb and Cd concentrations and lower Zn and Cu concentrations in females may be associated with SA and RPL. Exposure to toxic metals, as well as deficiencies in essential metals, may cause SA and RPL through endocrine dysfunction, such as insulin resistance, vitamin D insufficiency, and abnormal thyroid and sex hormone concentrations. However, further prospective cohort and experimental studies are required to provide stronger evidence.

## Data availability statement

The original contributions presented in the study are included in the article/**Supplementary Material**. Further inquiries can be directed to the corresponding authors.

## Author contributions

XS and MR proposed the subject and designed the protocol for this systematic review. MR, LTW, LQW and JC conducted literature screening and data extraction. MR, LQW, and LTW assessed the quality of all studies. LTW and MR performed statistical analysis. MR, LTW, and LQW produced the tables, figures. MR, LQW and LTW drafted the manuscript. XS and SQ gave overall supervision, critical revisions, and final approval of the article. All authors listed have made a substantial, direct, and intellectual contribution to the work and approved it for publication.

## Supplementary material

Supplementary Figure 1 Subgroup analysis of circulating Zn level in abortion women or healthy pregnant women. **(A)**. Subgroup analysis based on the follow-up endpoint (ongoing pregnancy and live birth); **(B)**. Subgroup analysis based on the continent of study population (Africa, Asia, Europe, and Oceania); **(C)**. Subgroup analysis based on the type of article (case-control study, cross-section study, and nested case-control study); **(D)**. Subgroup analysis based on the type of detected sample (serum, plasma, and whole blood).

Supplementary Figure 2 Subgroup analysis of circulating Cu level in abortion women or healthy pregnant women. **(A)**. Subgroup analysis based on the follow-up endpoint (ongoing pregnancy and live birth); **(B)**. Subgroup analysis based on the continent of study population (Africa, Asia, Europe, and Oceania); **(C)**. Subgroup analysis based on the type of article (case-control study, cross-section study, and nested case-control study); **(D)**. Subgroup analysis based on the type of detected sample (serum, plasma, and whole blood).

Supplementary Figure 3 Subgroup analysis of circulating Pb level in abortion women or healthy pregnant women. **(A)**. Subgroup analysis based on the follow-up endpoint (ongoing pregnancy and live birth); **(B)**. Subgroup analysis based on the continent of study population (Africa, North America, Asia, and Europe); **(C)**. Subgroup analysis based on the type of article (case-control study and cross-section study); **(D)**. Subgroup analysis based on the type of detected sample (serum, plasma, and whole blood).

Supplementary Figure 4 Subgroup analysis of circulating Cd level in abortion women or healthy pregnant women. **(A)**. Subgroup analysis based on the follow-up endpoint (ongoing pregnancy and live birth); **(B)**. Subgroup analysis based on the continent of study population (Africa, Asia, and Europe); **(C)**. Subgroup analysis based on the type of detected sample (serum and whole blood).
