# Supplementary figures and images for: Association between female circulating heavy metal concentration and abortion: a systematic review and meta-analysis
Source: Front Endocrinol (Lausanne). 2023 Aug 29;14:1216507. doi: 10.3389/fendo.2023.1216507 (PMC10497972; doi:10.3389/fendo.2023.1216507)

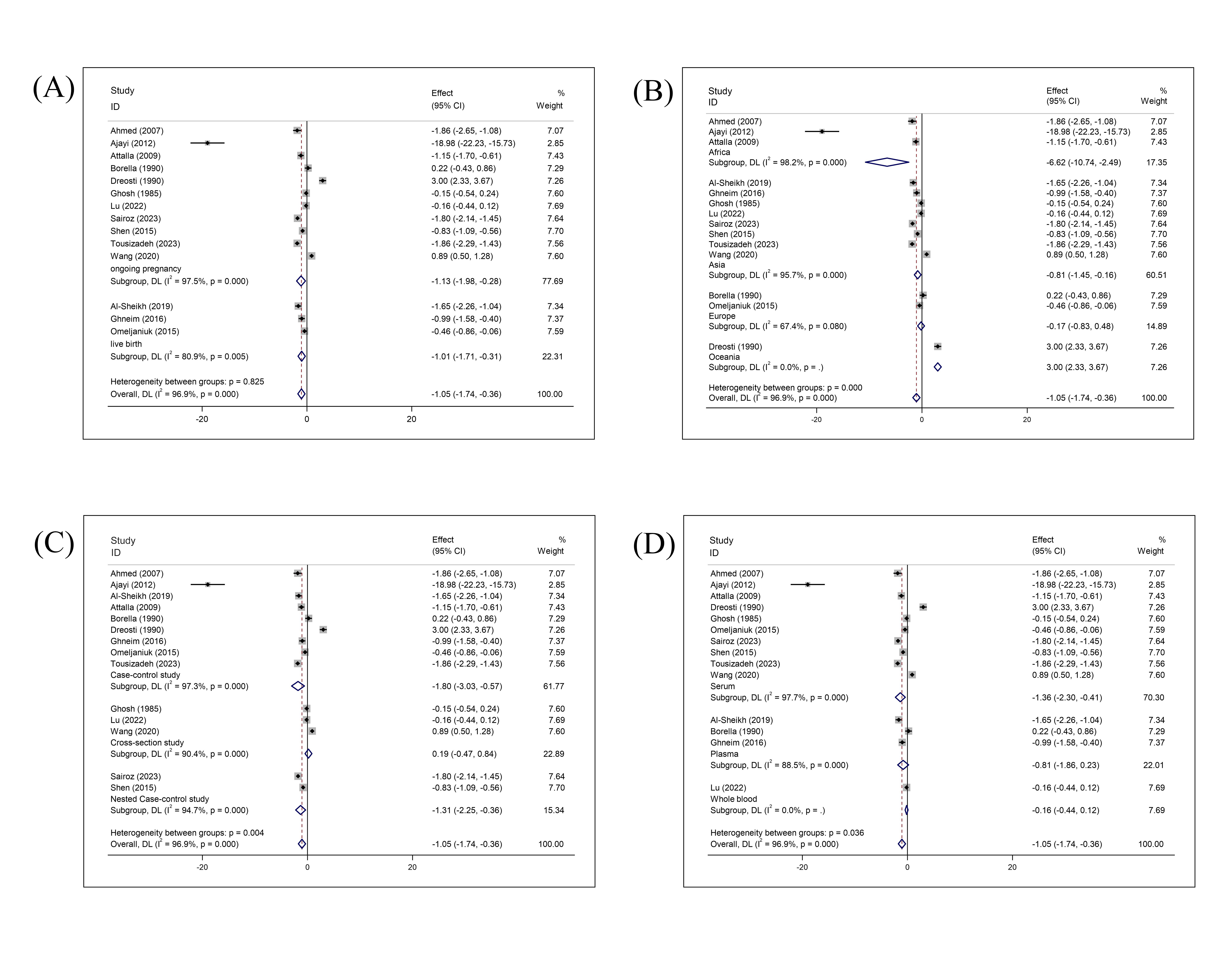

Supplement: Supplementary Figure 1 — Subgroup analysis of circulating Zn level in abortion women or healthy pregnant women. (A). Subgroup analysis based on the follow-up endpoint (ongoing pregnancy and live birth); (B). Subgroup analysis based on the continent of study population (Africa, Asia, Europe, and Oceania); (C). Subgroup analysis based on the type of article (case-control study, cross-section study, and nested case-control study); (D). Subgroup analysis based on the type of detected sample (serum, plasma, and whole blood). [file Image_1.jpeg]

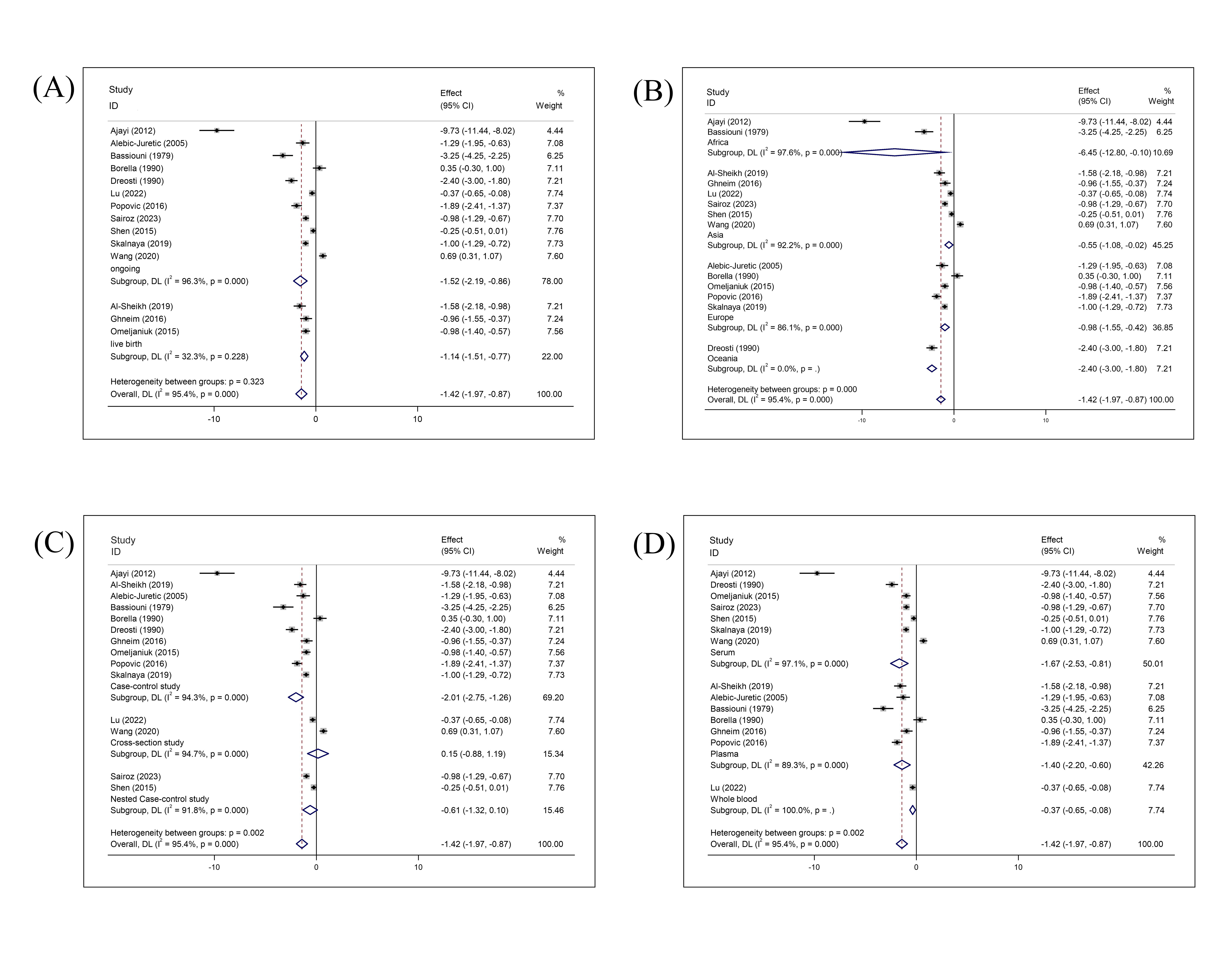

Supplement: Supplementary Figure 2 — Subgroup analysis of circulating Cu level in abortion women or healthy pregnant women. (A). Subgroup analysis based on the follow-up endpoint (ongoing pregnancy and live birth); (B). Subgroup analysis based on the continent of study population (Africa, Asia, Europe, and Oceania); (C). Subgroup analysis based on the type of article (case-control study, cross-section study, and nested case-control study); (D). Subgroup analysis based on the type of detected sample (serum, plasma, and whole blood). [file Image_2.jpeg]

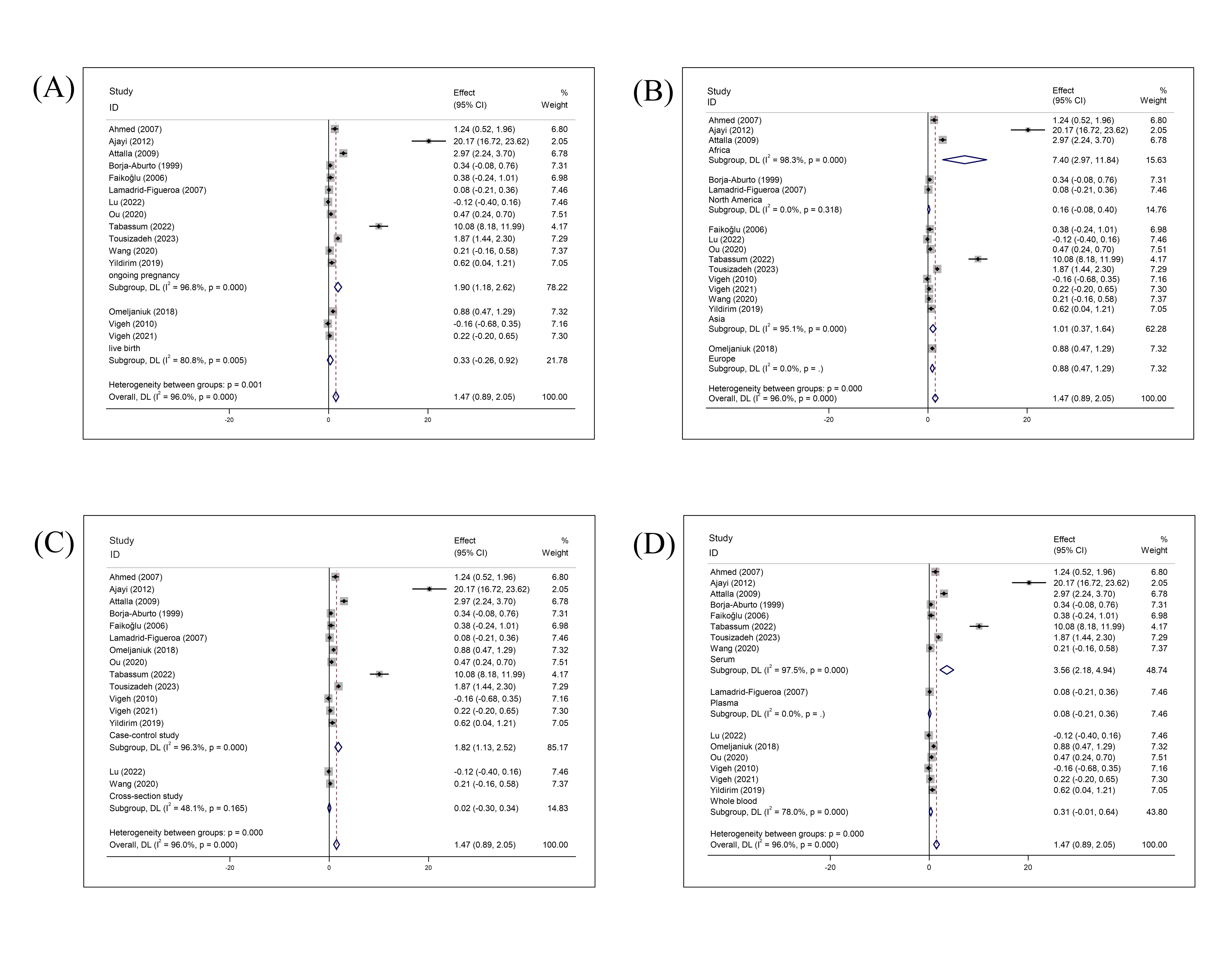

Supplement: Supplementary Figure 3 — Subgroup analysis of circulating Pb level in abortion women or healthy pregnant women. (A). Subgroup analysis based on the follow-up endpoint (ongoing pregnancy and live birth); (B). Subgroup analysis based on the continent of study population (Africa, North America, Asia, and Europe); (C). Subgroup analysis based on the type of article (case-control study and cross-section study); (D). Subgroup analysis based on the type of detected sample (serum, plasma, and whole blood). [file Image_3.jpeg]

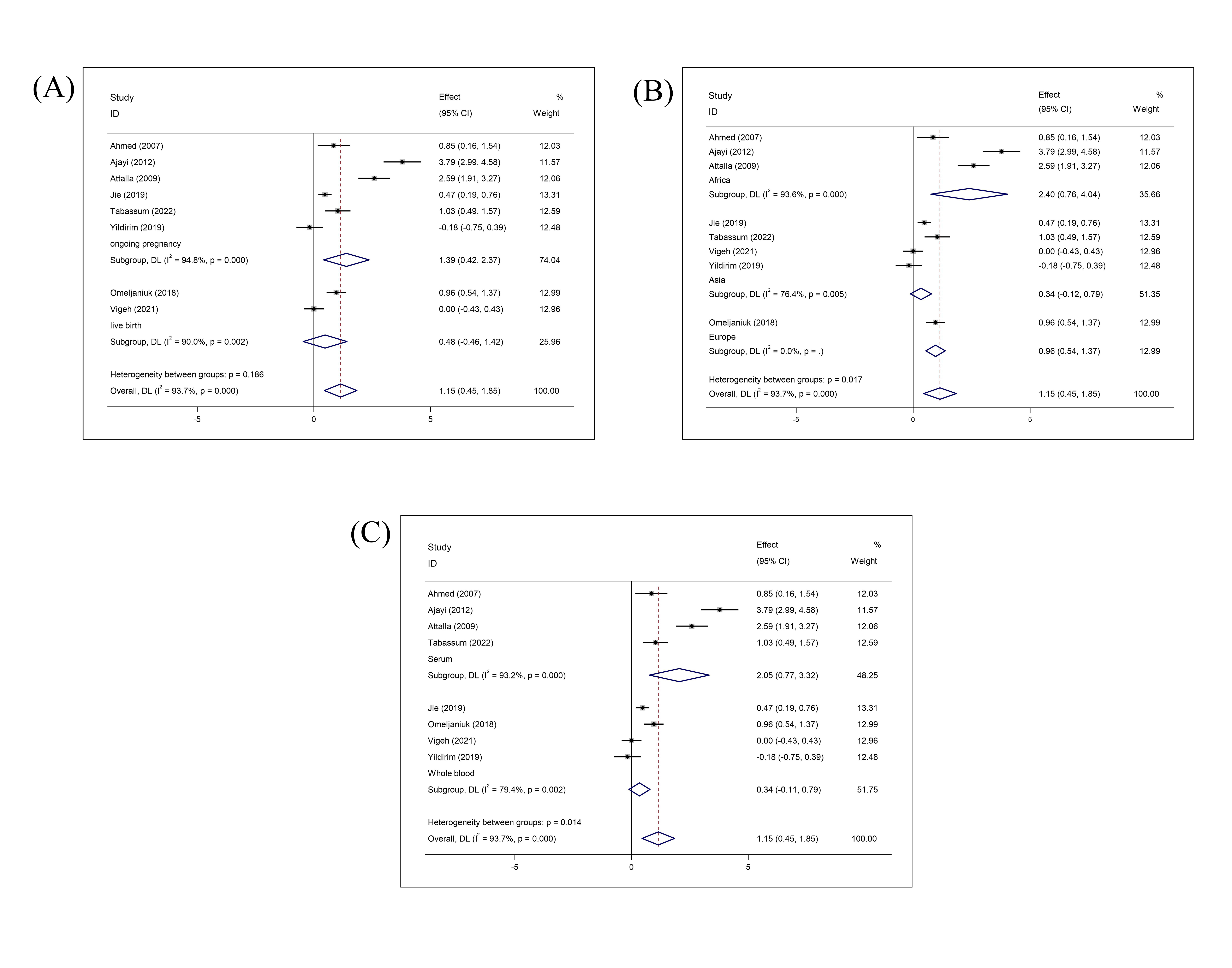

Supplement: Supplementary Figure 4 — Subgroup analysis of circulating Cd level in abortion women or healthy pregnant women. (A). Subgroup analysis based on the follow-up endpoint (ongoing pregnancy and live birth); (B). Subgroup analysis based on the continent of study population (Africa, Asia, and Europe); (C). Subgroup analysis based on the type of detected sample (serum and whole blood). [file Image_4.jpeg]
