# Supplementary material for: Association between female circulating heavy metal concentration and abortion: a systematic review and meta-analysis
Source: Front Endocrinol (Lausanne). 2023 Aug 29;14:1216507. doi: 10.3389/fendo.2023.1216507 (PMC10497972; doi:10.3389/fendo.2023.1216507)
Supplement: Supplementary file 5 [file Table_1.docx]

Supplementary Table 1 Newcastle–Ottawa Scale of included studies.

| **Sr.No** | **Author** | **Year** | **Selection** | | | |  | **Comparability** |  | **Exposure** | | | **Total scores** |
| --- | --- | --- | --- | --- | --- | --- | --- | --- | --- | --- | --- | --- | --- |
|  |  |  | **Adequate definition of case** | **Representativeness of the cases** | **Selection of controls** | **Definition of controls** |  | **Control for important factor** |  | **Ascertainment of exposure** | **Same method of ascertainment for cases and controls** | **Nonresponse rate** |  |
| 1 | Ahmed MH | 2007 | ★ | ★ | ★ | ★ |  | ★ |  | ★ | ★ | ★ | **8** |
| 2 | Ajayi, O. O. | 2012 | ★ | ★ | ★ | ★ |  | ★ |  | ★ | ★ | ★ | **8** |
| 3 | Alebic-Juretic, A. | 2005 | ★ | ★ | ★ | ★ |  |  |  | ★ | ★ | ★ | **7** |
| 4 | Al-Sheikh, Y. A. | 2019 | ★ | ★ | ★ | ★ |  | ★ |  | ★ | ★ | ★ | **8** |
| 5 | Attalla SM | 2009 | ★ | ★ | ★ | ★ |  | ★ |  | ★ | ★ | ★ | **8** |
| 6 | Bassiouni, B. A. | 1979 | ★ | ★ | ★ | ★ |  | ★ |  | ★ | ★ | ★ | **8** |
| 7 | Borella, P. | 1990 | ★ | ★ | ★ | ★ |  | ★ |  | ★ | ★ | ★ | **8** |
| 8 | Borja-Aburto, V. H. | 1999 | ★ | ★ | ★ | ★ |  | ★★ |  | ★ | ★ | ★ | **9** |
| 9 | Dreosti, I. E. | 1990 | ★ |  | ★ | ★ |  |  |  | ★ | ★ | ★ | **6** |
| 10 | Faikoǧlu, R. | 2006 | ★ | ★ | ★ | ★ |  | ★★ |  | ★ | ★ |  | **8** |
| 11 | Ghneim, H. K. | 2016 | ★ | ★ | ★ | ★ |  | ★ |  | ★ | ★ | ★ | **8** |
| 12 | Ghosh, A. | 1985 | ★ | ★ | ★ | ★ |  |  |  | ★ | ★ | ★ | **7** |
| 13 | Jie, O. | 2019 | ★ | ★ | ★ | ★ |  |  |  | ★ | ★ | ★ | **7** |
| 14 | Lamadrid-Figueroa, H. | 2007 | ★ | ★ | ★ | ★ |  | ★ |  | ★ | ★ | ★ | **8** |
| 15 | Lu, Y. | 2022 | ★ | ★ | ★ | ★ |  | ★★ |  | ★ | ★ | ★ | **9** |
| 16 | Omeljaniuk, W. J. | 2015 | ★ | ★ | ★ | ★ |  | ★ |  | ★ | ★ | ★ | **8** |
| 17 | Omeljaniuk, W. J. | 2018 | ★ | ★ | ★ | ★ |  | ★ |  | ★ | ★ | ★ | **8** |
| 18 | Ou, J. | 2020 | ★ | ★ | ★ | ★ |  | ★ |  | ★ | ★ | ★ | **8** |
| 19 | Popovic, J. K. | 2016 | ★ | ★ | ★ | ★ |  | ★★ |  | ★ | ★ | ★ | **9** |
| 20 | Sairoz | 2023 | ★ | ★ | ★ | ★ |  | ★ |  | ★ | ★ | ★ | **8** |
| 21 | Shen, P. J. | 2015 | ★ | ★ | ★ | ★ |  | ★ |  | ★ | ★ | ★ | **8** |
| 22 | Skalnaya, M. G. | 2019 | ★ | ★ | ★ |  |  | ★★ |  | ★ | ★ | ★ | **8** |
| 23 | Tabassum, Hajera | 2022 | ★ |  | ★ | ★ |  | ★ |  | ★ | ★ | ★ | **7** |
| 24 | Tousizadeh, S. | 2023 | ★ | ★ | ★ | ★ |  | ★★ |  | ★ | ★ |  | **8** |
| 25 | Vigeh, M. | 2010 | ★ | ★ | ★ | ★ |  | ★★ |  | ★ | ★ | ★ | **9** |
| 26 | Vigeh, Mohsen | 2021 | ★ | ★ | ★ | ★ |  | ★ |  | ★ | ★ | ★ | **8** |
| 27 | Wang, Ruixia | 2020 | ★ | ★ | ★ | ★ |  | ★ |  | ★ | ★ | ★ | **8** |
| 28 | Yildirim, E. | 2019 | ★ | ★ | ★ | ★ |  | ★★ |  | ★ | ★ | ★ | **9** |
