# Supplementary material for: Association between female circulating heavy metal concentration and abortion: a systematic review and meta-analysis
Source: Front Endocrinol (Lausanne). 2023 Aug 29;14:1216507. doi: 10.3389/fendo.2023.1216507 (PMC10497972; doi:10.3389/fendo.2023.1216507)
Supplement: Supplementary file 6 [file Table_2.docx]

**PubMed**: (Cadmium OR Lead OR Zinc OR Copper) AND ((Abortion, Habitual) OR (Habitual Abortion*) OR (Abortion, Recurrent) OR (Recurrent Abortion*) OR (Miscarriage, Recurrent) OR (Recurrent Miscarriage*) OR (Recurrent Early Pregnancy Loss)) OR ((Abortion, Spontaneous) OR (Abortions, Spontaneous) OR (Spontaneous Abortion*) OR (Early Pregnancy Loss*) OR (Loss, Early Pregnancy) OR (Losses, Early Pregnancy) OR (Pregnancy Loss, Early) OR (Pregnancy Losses, Early) OR (Miscarriage*) OR (Abortion, Tubal*) OR (Tubal Abortion*))

All 1473 articles were included.

**Embase**: #3 AND #4

#1: 'recurrent abortion'/exp OR 'abortion, habitual' OR 'abortion, recurrent' OR 'habitual abortion' OR 'repeated abortion' OR 'successive abortion'

#2: 'spontaneous abortion'/exp OR 'abortion, spontaneous' OR 'miscarriage'

#3: 'zinc'/exp OR 'copper'/exp OR 'lead'/exp OR 'cadmium'/exp

#4: #1 OR #2

All 334 articles were included.

**Cochrane Library**: #3 AND (#1 OR #2)

#1: (Abortion, Habitual) OR (Abortion, Recurrent) OR (Recurrent Early Pregnancy Loss) OR (Habitual Abortions) OR (Recurrent Abortion) OR (Recurrent Abortions) OR (Recurrent Miscarriage) OR (Habitual Abortion) OR (Recurrent Miscarriages) OR (Miscarriage, Recurrent)

#2: (Abortion, Spontaneous) OR (Miscarriages) OR (Miscarriage) OR (Spontaneous Abortions) OR (Abortions, Spontaneous) OR (Spontaneous Abortion) OR (Pregnancy Loss, Early) OR (Early Pregnancy Losses) OR (Early Pregnancy Loss) OR (Pregnancy Losses, Early) OR (Losses, Early Pregnancy) OR (Loss, Early Pregnancy) OR (Abortion, Tubal) OR (Tubal Abortions) OR (Abortions, Tubal) OR (Tubal Abortion)

#3: (Zinc) OR (Copper) OR (Cadmium) OR (Lead)

187 trials were included.

**Web of Science**: #3 AND (#1 OR #2)

#1: (Abortion, Habitual) OR (Habitual Abortion*) OR (Abortion, Recurrent) OR (Recurrent Abortion*) OR (Miscarriage, Recurrent) OR (Recurrent Miscarriage*) OR (Recurrent Early Pregnancy Loss)

#2: (Abortion, Spontaneous) OR (Abortions, Spontaneous) OR (Spontaneous Abortion*) OR (Early Pregnancy Loss*) OR (Loss, Early Pregnancy) OR (Losses, Early Pregnancy) OR (Pregnancy Loss, Early) OR (Pregnancy Losses, Early) OR (Miscarriage*) OR (Abortion, Tubal*) OR (Tubal Abortion*)

#3: Cadmium OR Lead OR Zinc OR Copper

2228 articles were included.
